# Laparoscopic Resection of a Gastric Diverticulum in an Adolescent

**DOI:** 10.7759/cureus.11161

**Published:** 2020-10-25

**Authors:** Jerry French, Arthur Meyers, Jennifer Mills, William Adamson, Tamarah Westmoreland

**Affiliations:** 1 Surgery, University of Central Florida College of Medicine, Orlando, USA; 2 Pediatric Surgery, Nemours Children's Hospital, Orlando, USA; 3 Pediatric Radiology, Cincinnati Children’s Hospital Medical Center, Cincinnati, USA; 4 Pediatric Surgery, University of Central Florida College of Medicine, Orlando, USA

**Keywords:** gastric diverticulum, pediatrics, surgery

## Abstract

Gastric diverticula rarely occur in adolescence. In adults, they are predominantly congenital, asymptomatic, and are located adjacent to the gastroesophageal junction on the posterior aspect of the stomach wall. In this report we present a 14-year-old female who underwent laparoscopic gastric diverticulectomy after incidental discovery on magnetic resonance urography.

## Introduction

Though gastric diverticula are uncommon in children, 4% of all the identified gastric diverticula are discovered in people less than 20 years of age. Recognition of this congenital malformation is helpful in identifying the correct treatment and avoid potential complications, such as ulceration or perforation [1]. In this report, we present a 14-year-old female who underwent laparoscopic resection of an incidentally discovered gastric diverticulum (GD) while being evaluated for abdominal pain.

## Case presentation

**Figure 1:**
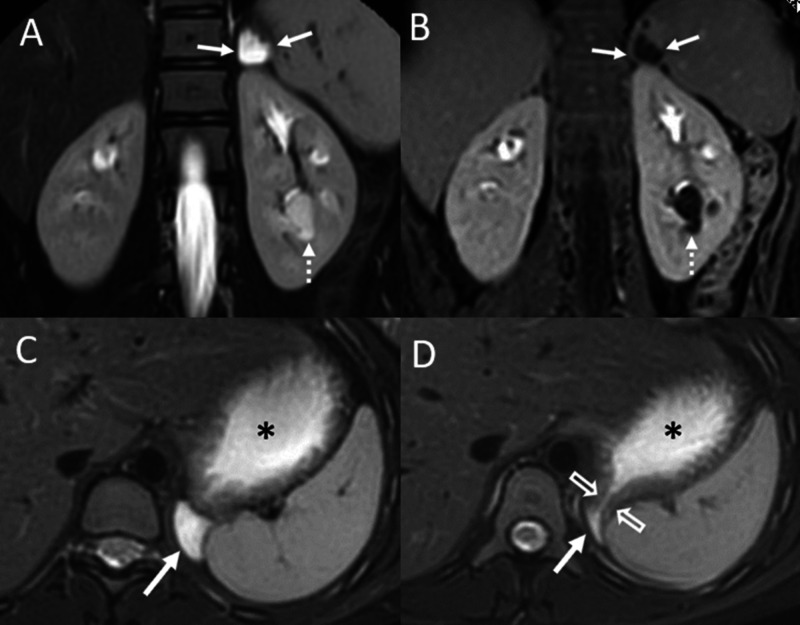
Gastric diverticulum diagnosed on an MR urogram. Coronal (A) T2-weighted and (B) T1-weighted contrast enhanced MR images show a left supra-renal lesion (solid arrows) that is increased signal intensity on the T2-weighted sequence (A) and shows peripheral enhancement on the post-contrast image (B). Consecutive axial T2-weighted images (C and D) show the close proximity of the lesion (solid arrows) to the stomach (asterisk) with the more superior image (D) showing a communication (open arrows) with the stomach. The dashed arrows (A and B) show a peri-pelvic left renal cyst.

**Figure 2:**
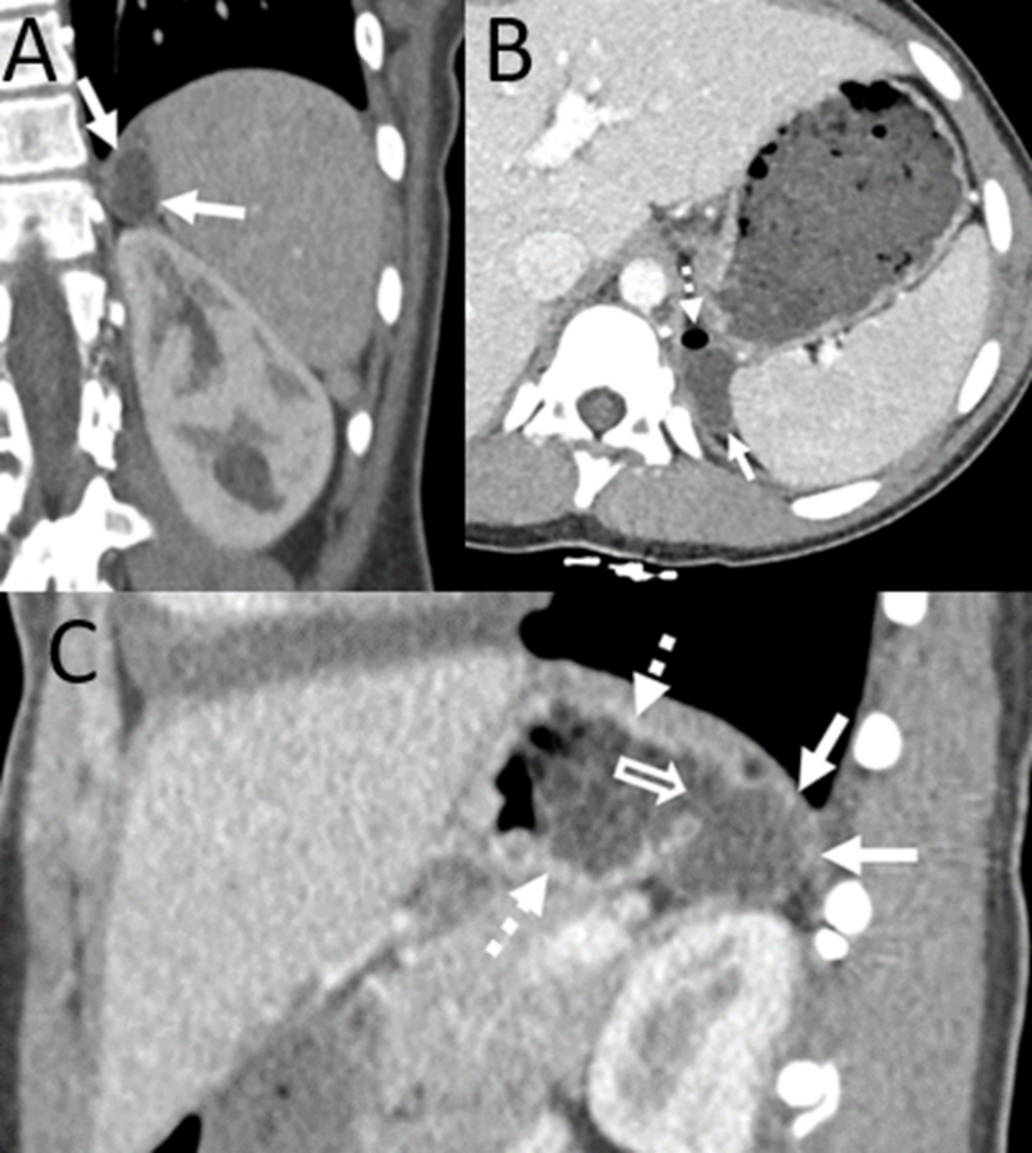
CT scan of the abdomen with IV contrast performed seven months earlier. Coronal (A), axial (B) and sagittal (C) images show the gastric diverticulum (solid arrows). Note the air along the non-dependent aspect of the diverticulum on the axial (B) image (dashed arrow). The sagittal image (C) clearly shows a connection (open arrow) between the diverticulum (solid arrows) and the fundus of the stomach (dashed arrows).

**Figure 3:**
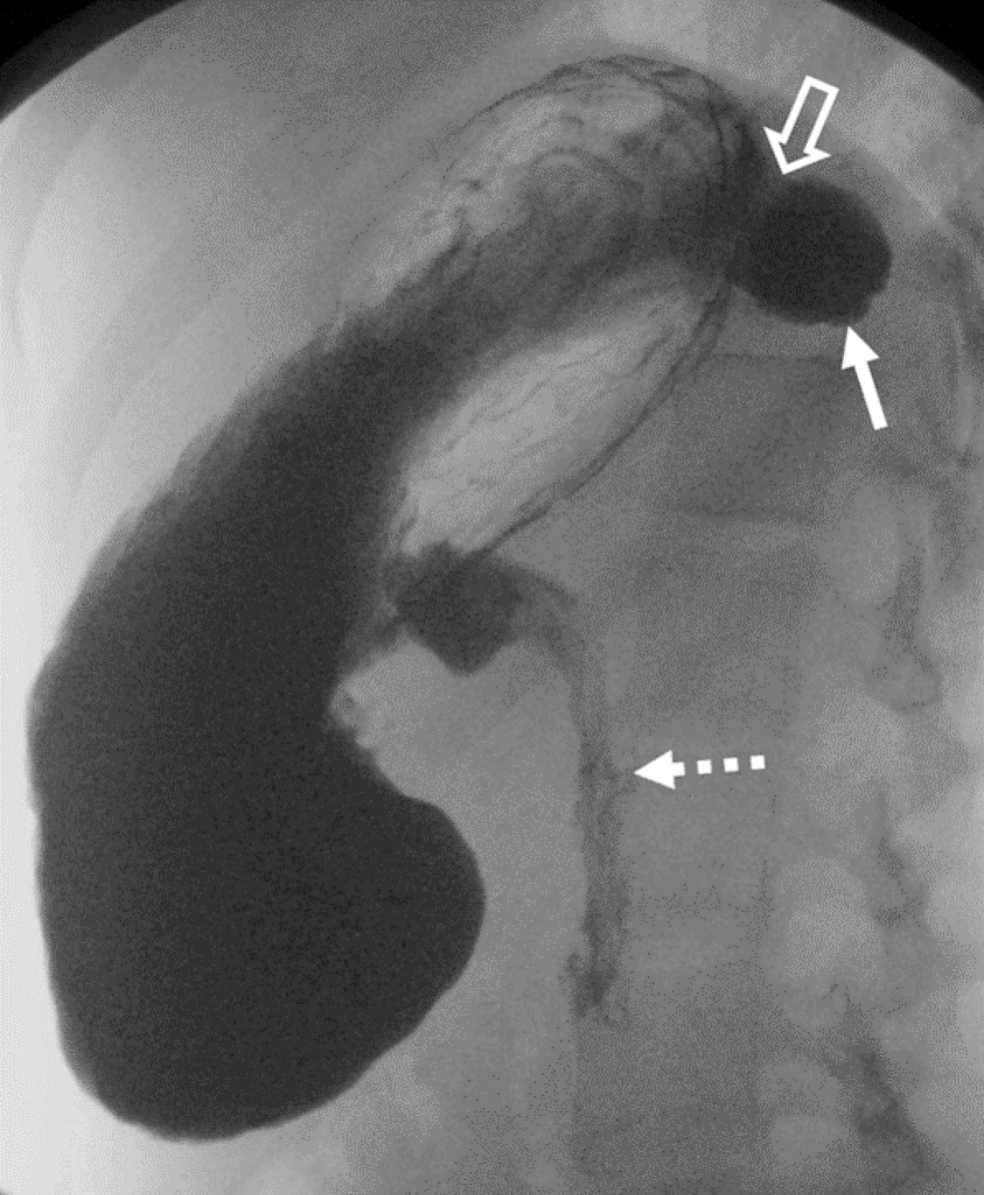
Upper GI exam performed on the same patient. Fluoroscopic image of the stomach after the administration of barium with the patient in a right lateral decubitus position. There is contrast filling the gastric diverticulum (solid arrow) and the connection with the posterior aspect of the stomach (open arrow) can be seen.  The dashed arrow points to contrast emptying into the duodenum for orientation purposes.

A 14-year-old female presented to our service following an incidental finding of a gastric diverticulum on magnetic resonance urography (Figure 1) while being followed for complex renal cysts. Review of images from a CT scan of the abdomen and pelvis performed seven months earlier for abdominal pain at an outside institution also showed the gastric diverticulum (Figure 2). However, this finding was not mentioned in the outside report for that CT scan. The patient had a yearlong history of intermittent abdominal pain and nausea without emesis. She denied any changes to her bowel habits during this time. Her father had a history of diabetes. Physical exam revealed a healthy, alert adolescent female in no acute distress. On palpation, her abdomen was soft, non-tender, and non-distended. A very small fascial defect was noted at the umbilicus, but no other masses or organomegaly were evident. She had normoactive bowel sounds on auscultation. A fluoroscopic upper gastrointestinal (UGI) exam showed a wide-mouth diverticulum arising from the posteromedial aspect of the gastric fundus (Figure 3). No extension of the diverticulum into the thoracic cavity was noted.

**Figure 4:**
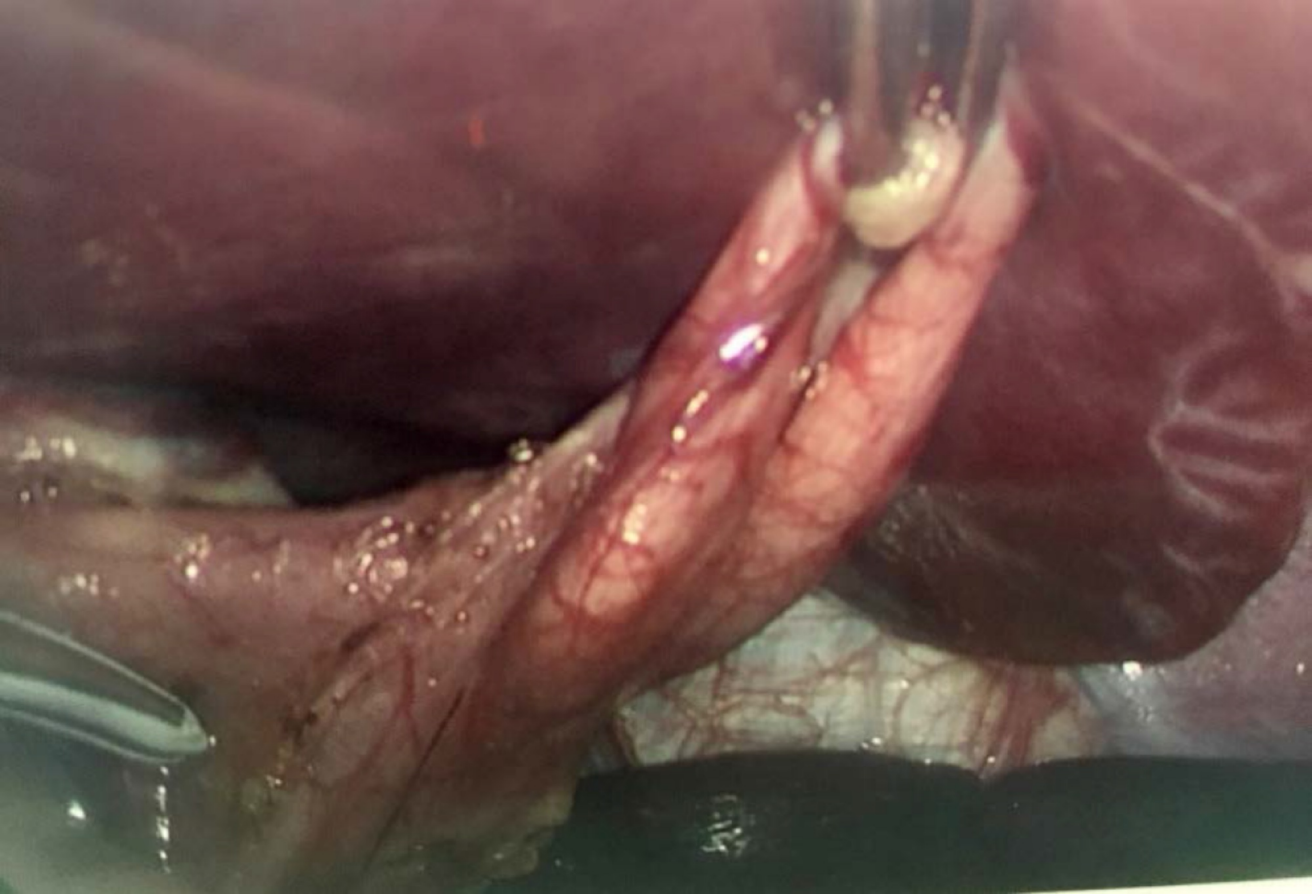
Intraoperative view of the gastric diverticulum.

The patient began omeprazole for her intermittent abdominal pain prior to the procedure and was instructed to continue the medication after surgery. In the operating room, a 2 cm infraumbilical incision was made, and electrocautery was used to progress through the subcutaneous tissue. The stalk of the umbilical hernia was isolated, opened, and a 5 mm port was inserted in the fascial defect. Carbon dioxide was infused into the abdominal cavity to a pressure of 15 mmHg. Three additional 5 mm ports were placed in the left and right upper quadrants and right mid-abdomen. The anterior surface of the stomach was free of abnormalities. The lesser sac was opened, and the short gastric vessels were divided using the LigaSure Maryland jaw device (Medtronic, Minneapolis, MN, USA). At the gastric fundus, approximately 2 cm distal from the gastroesophageal junction, the wide-mouth GD was identified (Figure 4). Of note, an endoscope was on standby in the operating room during the surgery to aid in its identification if necessary. The attachments to the GD were taken down using the LigaSure Maryland jaw device. The 5 mm umbilical port was replaced with a 12 mm port. The GD was divided away from the stomach using an Endo GIA reinforced stapler (Medtronic), placed in an Endo Catch bag (Medtronic), and removed from the abdomen through the umbilical port. The resected specimen was sent to pathology and found to have normally folded gastric mucosa with mild chronic inflammation. The patient was tolerating clear liquids on postoperative day one and was discharged after tolerating a regular diet on postoperative day two.

## Discussion

GD are rare compared to diverticula found in other locations throughout the gastrointestinal tract. The literature suggests the overall prevalence ranges from 0.01% to 0.11% in upper gastrointestinal endoscopies and up to 0.02% in autopsy studies [1]. It is estimated that 4% of cases occur in those under 20 years of age [2]. Approximately 70% of gastric diverticula are congenital. Congenital GD are predominantly located on the posterior aspect of the gastric cardia or fundus, whereas acquired GD are more often located in the gastric antrum. Acquired GD are false diverticula and can co-occur with gastric neoplastic disorders, peptic ulcer disease, and pancreatitis [3]. Most patients with GD are asymptomatic. If present, symptoms are nonspecific and may include abdominal pain, nausea, vomiting, early satiety, dyspepsia, and halitosis.

As was the case in our patient, GD are often discovered incidentally on cross-sectional imaging performed for other reasons but appears to be the source of her intermittent abdominal pain. When located posteriorly, GD seen on CT and MRI may be misinterpreted as left adrenal, splenic, or pancreatic tail pathologies [1]. This is particularly true when the connection between the stomach and the GD is decompressed and not visualized. Air bubbles, air-fluid levels, and oral contrast (if administered) within a GD have been reported as being helpful on CT to differentiate GD from adrenal lesions [1]. A fluoroscopic UGI with oral contrast is generally considered the most reliable imaging exam for the diagnosis of GD; however, they may go undetected on UGI if the neck is narrow and contrast does not enter the diverticulum [1]. Therefore, esophagogastroduodenoscopy (EGD) is still considered the gold-standard for the diagnosis of GD.

Asymptomatic diverticula do not require intervention; however, conservative treatment with proton pump inhibitors, histamine (H2) receptor antagonists, or antacids can provide temporary relief of symptoms [1]. In all forms of GD, surgery has been shown to alleviate symptoms in most of the reported symptomatic cases and prevents complications like that of any other type of gastrointestinal diverticula: ulceration, hemorrhage, perforation, obstruction, and malignancy [4]. GD with bases greater than 4 cm in diameter are at higher risk for complications and are more likely to be refractory to conservative therapy [5]. In open resections of GD near the gastroesophageal junction, superior mobilization of the fundus provides a more direct approach [3]. Laparoscopic resection is currently the preferred approach but is not without potential challenges, such as when the diverticulum is collapsed, hidden in the splenic bed secondary to its relationship to the short gastric vessels, or adhered to nearby tissue [1]. Proper preoperative planning, including having an endoscope ready for EGD, is essential for an uneventful surgery in case such challenges arise. It is possible that GD can arise as a complication of other surgical procedures on the stomach such as a Roux-en-Y gastric bypass [6].

## Conclusions

Gastric diverticula, while rare and often asymptomatic, can occur in children with vague abdominal symptoms. Cross-sectional imaging can detect and diagnose these lesions; however, at times they can be confused for other pathologies on these studies. Most GD can be diagnosed on UGI exams but ones with narrow necks may not be detectable if they do not fill with contrast. In such cases EGD can make the diagnosis by direct visualization. To avoid complications, laparoscopic resection is recommended for symptomatic cases or those with bases measuring more than 4 cm in diameter.
